# Organic Farming and Cover-Crop Management Reduce Pest Predation in Austrian Vineyards

**DOI:** 10.3390/insects12030220

**Published:** 2021-03-04

**Authors:** Jo Marie Reiff, Sebastian Kolb, Martin H. Entling, Thomas Herndl, Stefan Möth, Andreas Walzer, Matthias Kropf, Christoph Hoffmann, Silvia Winter

**Affiliations:** 1 Institute for Environmental Sciences, University of Koblenz-Landau, iES Landau, Fortstraße 7, D-76829 Landau in der Pfalz, Germany; kolb@uni-landau.de (S.K.); entling@uni-landau.de (M.H.E.); 2 Julius Kühn Institute, Federal Research Institute for Cultivated Plants, Institute for Plant Protection in Fruit Crops and Viticulture, Geilweilerhof, D-76833 Siebeldingen, Germany; christoph.hoffmann@julius-kuehn.de; 3 Institute of Plant Protection, University of Natural Resources and Life Sciences, Gregor-Mendel-Str. 33, A-1180 Vienna, Austria; thomas.herndl@students.boku.ac.at (T.H.); stefan.moeth@boku.ac.at (S.M.); andreas.walzer@boku.ac.at (A.W.); silvia.winter@boku.ac.at (S.W.); 4 Institute for Integrative Nature Conservation Research, University of Natural Resources and Life Sciences, Gregor-Mendel-Str. 33, A-1180 Vienna, Austria; matthias.kropf@boku.ac.at

**Keywords:** natural pest control, *Lobesia botrana*, grape berry moth, integrated vineyards, cover-crop management, sentinel cards, viticulture

## Abstract

**Simple Summary:**

Global declines in arthropods necessitate a rethinking of current agricultural practices. Organic farming, complex landscapes with high proportions of seminatural habitats and local vineyard management practices such as implementation of flower-rich cover-crop mixtures may be a promising approach to enhance arthropod biodiversity and, thus, natural pest control in viticulture. We examined effects of organic farming, different types of vineyard inter-row vegetation, and landscape composition on natural pest control of a major grapevine pest, the grape berry moth *Lobesia botrana*, and identified its dominant natural enemies. Surprisingly, natural pest control was reduced by sown cover-crops and organic farming. Interestingly, bush crickets were one of the most dominant natural enemies in the Austrian study region. Negative effects of organic farming in our study are most likely related to high fungicide inputs. Thus, a promising approach to reduce fungicide input and, therefore, promote a more sustainable viticulture may be the implementation of fungus-resistant grape varieties.

**Abstract:**

Habitat simplification and intensive use of pesticides are main drivers of global arthropod declines and are, thus, decreasing natural pest control. Organic farming, complex landscapes, and local vineyard management practices such as implementation of flower-rich cover-crop mixtures may be a promising approach to enhance predator abundance and, therefore, natural pest control. We examined the effect of organic versus integrated management, cover-crop diversity in the vineyard inter-rows, and landscape composition on the natural pest control of *Lobesia botrana* eggs and pupae. Predation of *L. botrana* pupae was reduced by organic farming and species-poor cover-crops by about 10%. Predation rates of *L. botrana* eggs did not differ significantly in any of the studied management options. Dominant predators were earwigs (Forficulidae), bush crickets (Tettigoniidae), and ants (Formicidae). Negative effects of organic viticulture are most likely related to the negative nontarget effects on arthropods related to the frequent sulfur and copper applications in combination with the avoidance of strongly damaging insecticides by integrated winegrowers. While a 10% difference in predation rates on a single pest stage is unlikely to have strong practical implications, our results show that the assumed effectiveness of environmentally friendly agriculture needs to be evaluated for specific crops and regions.

## 1. Introduction

Agricultural intensification, particularly habitat simplification, landscape homogenization, and intensive use of pesticides and fertilizers, is leading to global declines in arthropods [1,2,3], thus decreasing the provision of ecosystem services such as natural pest control [4,5]. Organic farming is a promising approach for enhancing natural enemies of pest species and, consequently, their effectiveness in pest control [6,7,8]. However, benefits of organic farming on pest control seem to be less prominent in perennial crops such as grapevine than in annual crops [9,10] and may depend on the landscape context [6,11]. Complex landscapes with higher proportions of seminatural areas often support higher levels of natural pest control [6,12,13]. Adjacent seminatural habitats, particularly woody vegetation, can promote natural enemies such as lacewings, spiders, predatory mites, rove beetles, and hymenopteran parasitoids in vineyards [14,15]. Nevertheless, adverse effects of landscape complexity on the occurrence of natural enemies, as well as on insect pest infestations, are also possible [16,17,18,19,20] and may be explained by higher resource supply in crops such as vineyards compared to the surrounding seminatural habitats [21,22]. In addition, during specific life stages, pests may also benefit from certain resources in seminatural habitats [21].

Furthermore, local vineyard management practices such as implementation of flower-rich cover-crop mixtures can enhance abundance of predatory mites, hymenopteran parasitoids, spiders, earwigs, and carabids [20,23,24]. Flower strips promote natural pest control in adjacent fields when tailored to the requirements of the target natural enemy taxa [25]. Thus, it can be expected that natural pest control is enhanced in vineyards sown with diverse cover-crop mixtures. However, it may be important to distinguish the effects of cover-crop mixtures relative to bare ground or relative to spontaneous vegetation in inter-rows as some authors showed higher arthropod diversity in vineyards with native ground cover [26]. Benefits of cover-crops over spontaneous vegetation have been studied less than their benefits relative to bare ground.

The European grapevine moth *Lobesia botrana* (Denis and Schiffermüller, 1775) (Lepidoptera: Tortricidae) is one of the major grapevine pests in Europe. In Europe, two to four generations of the polyphagous *L. botrana* [27] occur per year, with typically two (rarely three) generations occurring in Austria [28]. Larvae of the first generation feed on inflorescences, while those of the following generations damage berries, often resulting in *Botrytis cinerea* infections. Several taxa, including Dermaptera, Hemiptera, Neuroptera, Diptera, and Coleoptera, as well as ants and several families of spiders and mites, predate on *L. botrana* [29,30,31]. Furthermore, numerous parasitic Hymenoptera, particularly *Trichogramma* species and the ichneumonid *Campoplex capitatior*, attack different stages of *L. botrana* [32,33,34]. Several of these natural enemies are susceptible to fungicides such as sulfur and copper, both of which are frequently applied in organic viticulture instead of the predominant synthetic fungicides applied in conventional or integrated vineyards [35,36,37,38]. As fungicides are more effective when sprayed preventatively than curatively [39,40], they are the most frequently used pesticides in viticulture with several sprayings per year. There is growing demand from winegrowers for less harmful synthetic fungicides, which is reflected in comparably low nontarget effects of most of the synthetic fungicides [41]. Thus, it is unclear whether the widely observed benefits of organic farming to natural enemies and pest suppression [6] are present in current viticulture.

The aim of this study was to investigate the combined effects of organic farming, cover-crop management, and landscape composition on natural pest control of grape berry moths. We hypothesize that predation pressure on *L. botrana* is promoted by (i) organic compared to integrated management, (ii) diverse cover-crop mixtures, and (iii) heterogenous landscapes with high percentage of woody seminatural habitats. Furthermore, we aimed to identify the most important predators of *L. botrana* in Austrian vineyards.

## 2. Materials and Methods

### 2.1. Study Sites

**Figure 1:**
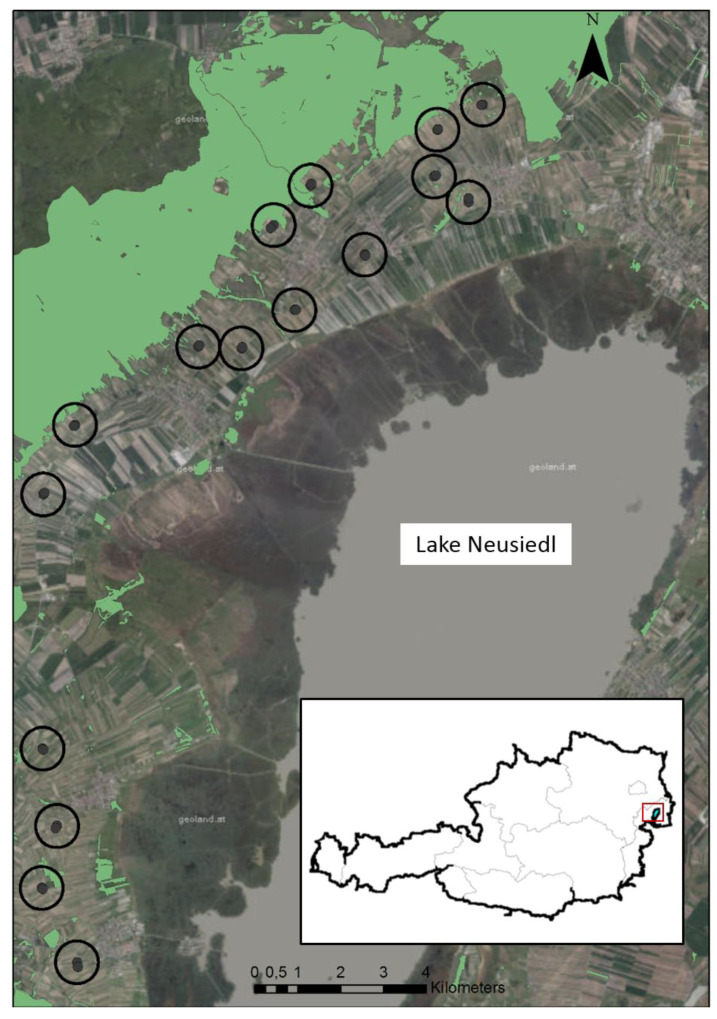
Study sites in eastern Austria.

We investigated 16 pairs of vineyards situated between the western shore of Lake Neusiedl and the Leithaberg hills (47°54′55″ north (N); 16°41′40″ east (E)) in eastern Austria (Figure 1). Each vineyard pair consisted of one vineyard under organic and one under integrated pest management. Inter-row management of cover-crops differed in both organic and integrated vineyards, resulting in three types of cover-crop management: species-rich cover-crop mixtures (20–34 species seeded), species-poor cover-crop mixtures (4–9 species), and spontaneous vegetation (no seeding of cover-crops for at least 5 years). The climate of the study region is warm temperate [42]; in 2019, the average temperature was 12.4 °C and total annual precipitation was 414 mm, which was warmer and dryer in comparison with the last 10 years (11.6 °C and 574 mm) [43]. While large continuous parts of the landscape consist of vineyards (Figure 1), winegrowers manage mostly small (0.25–1.5 ha; on average 0.74 ± 0.66 ha), long, and elongated units (hereafter “vineyards”) consisting of only a few rows which were organized in a trellis system.

In each vineyard, vegetation parameters were recorded in spring (April) and summer (July). Vegetation cover (%) was visually estimated in one inter-row per vineyard in four 1 × 1 m sub-plots. Vegetation height was measured 10 times in the corresponding row and the two adjacent inter-rows with the drop disc method [44] in spring and summer, using a round sanding pad with a surface of 0.04 m^2^ and a weight per unit area of 1.9 kg/m^2^. Vineyard management information (including spraying regimes and tillage practices) was collected through personal semistructured interviews with winegrowers of the studied vineyards. Grape berry moths were controlled with mating disruption pheromones in the study region. Area-related acute pesticide toxicity load contact (aAPTLc) for applied pesticides was calculated on the basis of contact acute median lethal dose (LD_50_) values for honeybees according to Möth et al. [45]. Management information for organic vs. integrated vineyards and different cover-crop managements are provided in Table 1 and Table 2 (for more detailed information, see Table S1, Supplementary Materials).

### 2.2. Predation Rate Assessment

To assess the pest control potential of grape berry moths, we exposed sentinel cards consisting of both eggs and pupae of *L. botrana*. For rearing of *L. botrana*, we followed the methods of Markheiser et al. [46]. Inside of the rearing containers, retainers were installed to allow oviposition on exchangeable polyethylene strips. Egg-laden strips were harvested after 24 h. Eggs were distributed relatively evenly, resulting in an average number of 52 ± 17 eggs per strip. Pupae were harvested from rearing cups. Five pupae were attached to each strip using duct tape (HEB19L10GC, TOOLCRAFT, Conrad Electronic SE, Hirschau, Germany). The remaining adhesive surface was covered with sand to prevent predators from sticking. Sentinel cards were stored at 4 °C until exposure.

To determine predation rates, sentinel cards were attached to randomly selected 1 year old branches and were exposed there for 72 h. We exposed five sentinel cards per vineyard four times between June and August 2019 (3 June, 1 July, 30 July, 26 August) aligning with four phenological stages of the vines (full flowering, berry touch, veraison, and before harvest). The numbers of eggs and pupae were counted before and after exposure (for more detailed information, see Table S1, Supplementary Materials).

To determine the most important predators of *L. botrana* eggs and pupae, additional sentinel cards were exposed, and predation was monitored with four cameras in one of the vineyard pairs (one organic and one integrated). This was repeated eight times between June and August of 2019, resulting in 64 camera observations. We used a camera system consisting of a raspberry pi computer (Raspberry Pi Foundation, UK) and a camera module with two infrared light-emitting diodes (IR-LEDs; Electreeks, Dresden, Germany). A power-bank (Voltcraft PB-19) allowed the camera system to run for 24 h in the vineyards. The camera systems were placed in plastic containers to protect them from moisture and mounted on wooden poles which were driven into the ground with a distance of about 30 cm between cameras and sentinel cards. The cameras were programmed to take a picture every 10 s for 24 h.

### 2.3. Landscape Analysis

Landscape composition was analyzed in 500 m buffers around each studied vineyard. According to the available base maps and field mapping, land use was categorized following the EUNIS (European Nature Information System) habitat type classification [47] and later digitized in ArcGIS 10.6 [48]. Relative proportions of habitat types were calculated in ArcGIS 10.6, whereas landscape indices were computed with the R package “landscapemetrics” [49]. Due to our paired design, landscape composition was very similar in each pair of organic and integrated vineyards. The “average” landscape composition was characterized by 60.5% ± 16.6% agricultural area, of which 44.3% ± 15.7% was planted with vineyards, and 29.4% ± 13.7% seminatural habitats, of which 13.4 ± 15.0% were woody. The average distance of the studied vineyards to the next woody seminatural habitat was 23.4 ± 19.3 m, and the Shannon landscape diversity index was 1.49 ± 0.23.

### 2.4. Data Analysis

We summed predation data over all sampling dates and replicates per vineyard, resulting in one observation for each vineyard. All statistical analyses were done in R version 3.6.3 [50]. The distribution of response and predictor variables was checked visually using “qqp” (R package car) [51]. Multicollinearity among these predictor variables was evaluated by calculation of correlation matrices (function “corrplot”, R package corrplot) [52]. Correlation of predictor variables with factorial management options (organic/integrated; cover-crop management) was evaluated using linear and generalized linear models according to distribution of response variables (functions “lm” and “glm”). Summer vegetation and bare ground cover, amount of compost and artificial fertilizer, number of applications with pesticides in general, sulfur, copper, synthetic fungicides, insecticides, and herbicides, as well as aAPTLc, were correlated with the farming type and were, therefore, not included in the modeling process (compare Table 1). Effects on egg and pupae predation rates were analyzed using linear mixed-effect models (function “lmer”, package lme4) [53]. Models contained “vineyard pair” as a random factor and “cover-crop” (species-poor/species-rich/spontaneous), “management” (organic/integrated), and “proportion of woody seminatural habitats” as fixed predictor variables. A post hoc test was performed to differentiate significances in the variable “cover-crop” (“Tukey”, function “glht”, package multcomp) [54]. Assumptions were checked for all models using graphical validation procedures [55].

## 3. Results

**Figure 2:**
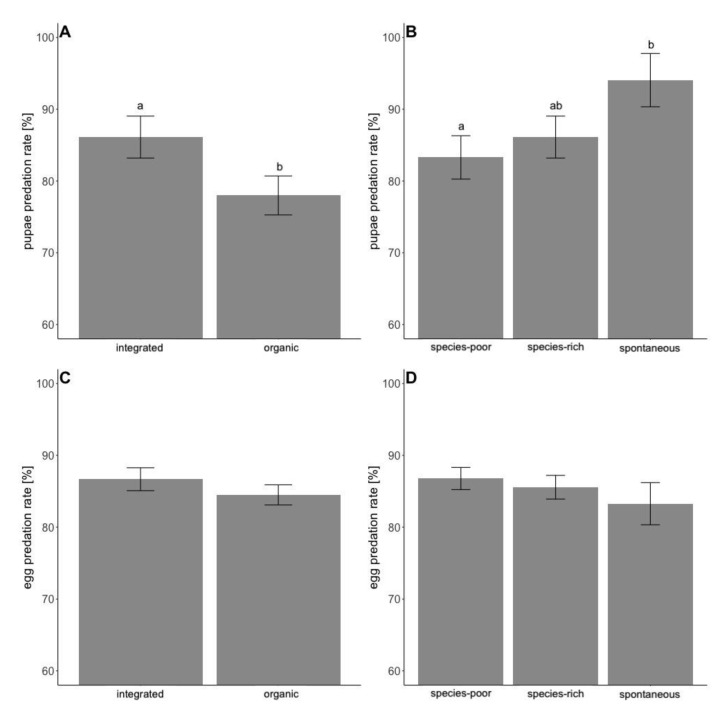
Differences in predation rates of *L. botrana* pupae (**A**,**B**) and eggs (**C**,**D**) between management systems (**A**,**C**) and implementation of cover-crops (**B**,**D**). Model predicted means and standard errors are displayed. Significant differences according to Tukey test are indicated by different letters.

**Figure 3:**
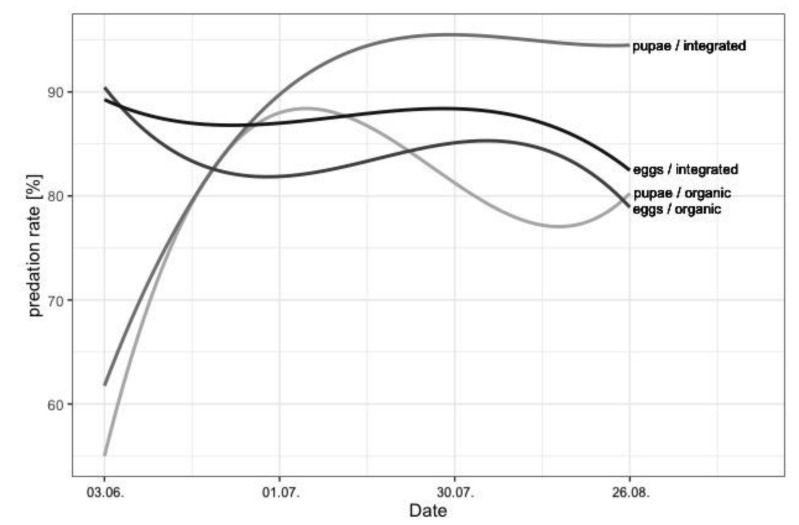
Mean predation rates of *L. botrana* pupae and eggs in organic and integrated vineyards for four sampling dates (3 June 2019, 1 July 2019, 30 July 2019, 26 August 2019).

On average, 85.6% of *L. botrana* eggs and 82.5% of *L. botrana* pupae were predated during the sampling period. Contrary to our expectations, predation of L. *botrana* pupae was increased on average by 10% in integrated compared to organic vineyards (Table 3, Figure 2A). Furthermore, predation was 10% higher in vineyards with spontaneous vegetation compared to species-poor cover-crops, with species-rich cover-crops showing intermediate values (Table 3, Figure 2B). Landscape composition had no clear effect on the predation of *L. botrana* pupae. Predation of *L. botrana* eggs was not influenced by vineyard management, implementation of cover-crops, or landscape composition (Table 3, Figure 2C,D). Predation rates of *L. botrana* pupae increased during summer, while egg predation rates slightly decreased during the same period (Figure 3).

**Figure 4:**
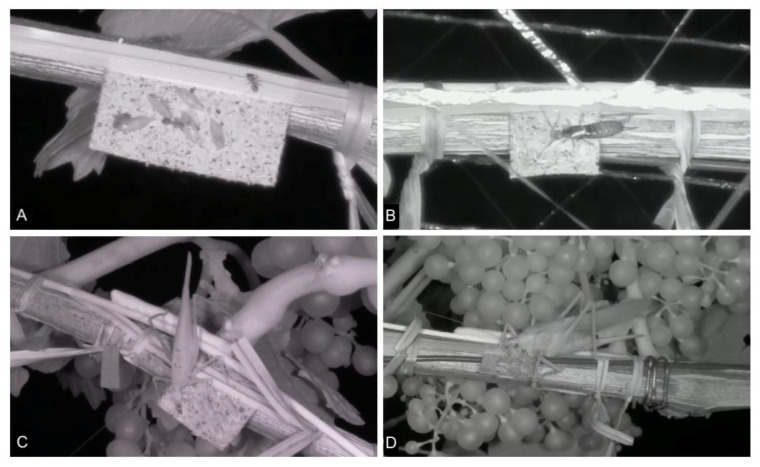
Predators photographed on sentinel cards. Selected pictures of (**A**) Formicidae, (**B**) *Forficula auricularia*, (**C**) *Phaneroptera nana* (male), and (**D**) *Tettigonia viridissima* (female).

We observed 455 arthropod individuals on the exposed sentinel cards, of which 178 individuals of 18 taxa actively predated or parasitized *L. botrana*. Predation and parasitism mainly occurred at dusk or throughout the night (88% nocturnal pupae predation, 60% nocturnal egg predation/parasitism; Table 4). Dominant predators were earwigs (Forficulidae), bush crickets (Orthoptera: Tettigoniidae), and ants (Formicidae). While ant densities were higher in organic than in integrated vineyards, densities of earwigs were higher in integrated vineyards, which was in accordance with the positive effect of integrated farming on *L. botrana* pupae predation (Table 4). For Orthoptera, more individuals were recorded in the organic vineyard, but the number of species was equal in both management types (*n* = 4). Selected photographs of the most frequent predators are displayed in Figure 4.

## 4. Discussion

Pupae predation increased over the season from a range of 55–62% during full flowering of grapevine to 80–95% before harvest. The increase in canopy architecture in the course of this vegetation period offers more habitat for natural enemies [56,57]. Furthermore, in correspondence to their life cycle, several natural enemies are more active during summer than during spring (e.g., bush crickets). A slight decrease in predation rates in early July and late August could be observed for both *L. botrana* pupae and eggs, which may be correlated to high temperatures during August. Hot weather conditions may impact parasitoids and predators and result in changes in feeding niches [58,59]. Nevertheless, predation rates were generally relatively high with about 80% for pupae and 85% for eggs. For comparison, Pennington et al. [29] detected *L. botrana* egg predation rates of about 35% in Germany, while, in France, on average 42% of eggs and 86% of larvae were predated [11]. The difference in predation rates could be attributed to either the landscape-scale usage of mating disruption pheromones against grape berry moths in the Austrian study region (i.e., covering both organic and integrated vineyards for over 10 years), thereby reducing the insecticide use drastically, whereas, in Bordeaux, insecticides were applied both in organic and in conventional vineyards [11]. Furthermore, the small size of the elongated vineyards in our study region consisting only of a few vine rows could lead to a larger contact area between vineyards and surrounding seminatural areas (i.e., stronger edge effects), which could facilitate natural pest control in comparison to larger, more quadratic shaped vineyards in Bordeaux [11] and the Palatinate winegrowing region [29]. The positive influence on pest control in elongated fields with a higher perimeter-to-area ratio was also shown in other studies for annual cropping systems [4,60,61]. This effect can be attributed to shorter distances from edges to fields and a larger contact zone between crop fields and adjacent seminatural habitats.

Counterintuitively, organic farming did not enhance natural pest control, but actually reduced pupae predation rates. This can be explained by the higher number of pesticide applications in organic vineyards that include a high frequency of nonspecific fungicides such as copper and sulfur, resulting in aAPTLc values that were twice as high as those in integrated vineyards (see Table 1). Sulfur is well known for its negative effects on a wide variety of natural enemies in viticulture with one of the highest IOBC (International Organisation for Biological and Integrated Control) toxicity ratings [38]. Fungicide applications with copper, sulfur, and potassium bicarbonate also reduced the number of natural enemies and egg predation in Germany [29]. Moreover, Muneret et al. [11] found increased egg predation but unaltered larval predation on grape berry moths with decreasing pesticide use, although pesticide use was lower in organic compared to conventional vineyards in France. Pesticide input was twice as high in conventional compared to organic vineyards in this French study [19]. This difference in pesticide input in organic vs. conventional/integrated vineyards could be related to different pesticide use in Austria and France. In Bordeaux, 6% of the applied pesticides were insecticides against grape berry moths and leafhoppers [19], whereas, in Austria, insecticides were only applied once in two vineyards. In general, fungicides are the most commonly applied pesticides in viticulture, representing 96% of all applications [62]. Due to the fact that mainly pesticide application frequency led to differences between organic and conventional viticulture in our study, we highlight the impact of fungicide toxicity to nontarget organisms. Reduced pesticide applications and/or implementation of less harmful substances are a promising approach to enhance natural pest control in vineyards [10,29] and should be considered in agri-environmental schemes.

We detected the highest pupae predation rates in vineyards with spontaneous vegetation cover. Contrastingly, *L. botrana* egg predation rates were higher in Germany in an experimental vineyard sown with flower-rich cover-crops compared to spontaneous vegetation [33]. Native cover-crop species enhanced arthropod diversity in South African vineyards [26] and natural enemy abundance and predation on light brown apple moth in Australia [63]. Furthermore, predator abundances of spiders, earwigs, and ants are negatively affected by tillage intensity [24,64,65]. Moreover, higher *L. botrana* infestations were recorded in vineyards which were frequently tilled compared to those planted with cover-crops in Italy [65]. In our study, bare soil vineyards were absent, but tillage intensity was lowest in vineyards with spontaneous vegetation, which could also explain the higher predation rates in those vineyards. Furthermore, herbicides were only applied in four integrated vineyards with species-poor cover-crops (only underneath the vines). Tillage frequency in the rows was higher in organic vineyards. These disturbance factors might have affected predator occurrence and pest control in our study. Possible benefits of diverse spontaneous vegetation or flower-rich cover-crops which provide resources for predatory arthropods should be considered in agri-environmental schemes in viticulture.

Unexpectedly, neither egg predation nor pupae predation was affected by landscape composition. Similarly, *L. botrana* infestation and predator abundance were not affected by proportion of seminatural habitats in France [19,64], while *L. botrana* egg predation even decreased with increasing proportion of seminatural habitats [11]. In the same region, Papura et al. [31] showed that harvestmen (which are important predators of grape berry moths) populations increased with increasing proportions of seminatural habitats at the landscape scale. Gonçalves et al. [66] observed a general decrease in predator abundance with increasing proportions of seminatural habitats. In the same study, ant abundance in vineyards increased with an increasing proportion of large-scale vineyards (500–750 m radius) and small-scale seminatural habitats (125–250 m radius). Contrarily, Thomson and Hoffmann [15] found increased abundance of natural enemies and parasitism of pest moth eggs in vineyards adjacent to woody vegetation in Australia. We assume that perennial crops such as vineyards provide enough food resources and shelter for agrobiont species such as earwigs and ants to accomplish their whole life cycle within the field, mitigating the effects of surrounding landscapes [66,67,68,69]. Spillover effects between seminatural habitats and crops that are observed in annual crops may be less relevant in vineyards [70]. In particular, when diverse vegetation cover is implemented in vineyard inter-rows, landscape effects may become negligible [20,71]. 

Even though the pictures of natural enemies of *L. botrana* were taken in only two of the studied vineyards and are, therefore, not representative for the whole study region, they provide valuable insights into the range of natural enemies of *L. botrana*. We captured 178 natural enemies predating actively on *L. botrana* in photographs, highlighting particularly the pest control potential of earwigs, ants, and bush crickets. Camera observations from vineyards in New Zealand revealed highest predation activities in grapevine canopies from earwigs [72]. Similarly, Pennington et al. [29] confirmed *L. botrana* egg predation by ants, earwigs, and green lacewings with camera-surveilled sentinel cards. Interestingly, we detected the highest activity of natural enemies at dusk and at night, which agrees with the results of Frank et al. [72], finding 50–60% nocturnal predation activity.

Earwigs prey on lepidopteran pest eggs, larvae, and pupae [72,73], but may be problematic in vineyards when occurring at high densities due to possible feeding on grape berries, contamination of grape bunches with feces, and spread of fungal pathogens, which decrease the must quality of the grapes [74,75]. Although earwigs can fly, their abundance on cherry trees was limited by isolation from other woody habitats [76]. In contrast, earwig abundance was affected neither by plant species richness or landscape composition nor by organic vs. conventional management in apple orchards [67]. However, pesticide reduction in apple orchards enhanced earwigs and other natural enemies and, thus, the control of woolly aphids [77]. In accordance with their reliance on ground vegetation during their early life stages, earwigs were more abundant in Spanish vineyards with spontaneous vegetation compared to bare soil and sown flower-rich cover-crops [24]. In our study, integrated vineyards received less fungicides and, therefore, had lower toxicity values, which accordingly promoted earwig occurrence and *L. botrana* predation. Thus, higher earwig occurrence and, correspondingly, more natural pest control can be expected in vineyards with minimal disturbance in terms of soil tillage and pesticide load.

Ants can impede the biological control of mealybugs and aphids, protecting them from natural enemies while feeding on their honeydew [65,78]. Due to this fact, ants are undesired in some winegrowing regions. Nevertheless, native ant communities did not affect other arthropod predators and predation rates on moth pests in Australian vineyards [79]. In addition, ants are major natural enemies of various pests, including moths, in blueberry, corn, and grasslands in the United States (US) [80]. Ant densities were not influenced by landscape compositions in cherry orchards in Switzerland or in vineyards in Portugal [66,81], but abundances were increased in organic vs. conventional vineyards and under implementation of cover-crops vs. tillage in Italy [65,82]. Ants were also more prevalent on the recorded sentinel cards in the organic vineyards in this study, which could be attributed either to ants’ higher tolerance toward pesticide toxicity or to avoidance strategies, as seen by higher earwig abundance in integrated vineyards.

Many bush crickets, such as the two *Phaneroptera* species observed in this study as the most dominant predators of *L. botrana* pupae (Table 4), are omnivores that can feed both on (young) plant parts and on pests of grapevine [83,84,85]. Other bush crickets (*Meconema meridionale*, *M. thalassinum*, and *Tettigonia viridissima*) are almost exclusively carnivorous [86] and known to prey on pests such as leaf miners, chrysomelids, and green leafhoppers [87,88,89]. Interestingly, our study is the first to show the large potential of bush crickets for grape berry moth control in Austrian vineyards, whereas bush crickets themselves are sometimes considered pests in other regions such as California [84,85].

## 5. Conclusions

We found small but positive effects of spontaneous vegetation and integrated farming on natural pest control of *L. botrana* pupae in Austrian vineyards. The comparably negative effect of organic viticulture is most likely related to the negative nontarget effects of frequent sulfur (and copper) applications on arthropods in combination with the avoidance of strongly damaging insecticides by integrated winegrowers. The long-term use of mating disruption pheromones against the grape berry moths has greatly reduced the necessity of insecticide use in the study region. This shows that management practices such as organic farming, which are often considered positive for biodiversity and pest control, need to be judged on a case-by-case basis. Furthermore, fungicide use also varies within organic and integrated vineyards, indicating a potential for fungicide reduction. New, fungus-resistant varieties offer an interesting opportunity for more sustainable viticulture by allowing fungicide reductions of up to 95% [57]. Furthermore, agri-environmental schemes (e.g., Austrian agri-environmental program) should promote the use of spontaneous vegetation cover or species-rich cover-crop mixtures to increase biodiversity and associated ecosystem services such as natural pest control in vineyards.

## Figures and Tables

**Table 1:** Treatment variables of organic and integrated vineyards (mean ± standard deviation). Bold numbers display variables that are significantly different between organic and integrated management. aAPTLc = area-related acute pesticide toxicity contact load. Tillage frequency differed in neighboring inter-rows; therefore, management measures are displayed for the inter-row in which vegetation data were collected in spring and summer.

	Organic	Integrated
aAPTLc	**19.95 ± 6.88**	**10.61 ± 14.93**
Total number of pesticide applications	**9.69 ± 1.74**	**6.31 ± 2.30**
Copper applications	**8.88 ± 1.89**	**1.25 ± 0.86**
Sulfur applications	**9.19 ± 1.64**	**4.13± 1.89**
Applications with synthetic fungicides	–	**5.75 ± 2.67**
Insecticide applications	–	**0.25 ± 0.45**
Acaricide applications	0.25 ± 0.45	0.38 ± 0.50
Herbicide applications	–	**0.31 ± 0.60**
Compost fertilizer (m^3^/ha)	**3.28 ± 5.06**	**0.63 ± 2.50**
Artificial fertilizer (kg/ha)	–	**31.25 ± 68.01**
Tillage frequency in inter-rows	1.00 ± 0.97	1.19 ± 1.47
Tillage frequency in rows	2.81 ± 0.83	2.50 ± 1.51
Years without tillage in inter-rows	**2.63 ± 1.40**	**2.31 ± 1.99**
Mulching frequency in inter-rows	2.00 ± 1.32	2.13 ± 0.96
Rolling frequency in inter-rows	0.44 ± 0.81	0.81 ± 1.33
Inter-row vegetation height in spring (cm)	11.45 ± 9.90	10.70 ± 8.97
Row vegetation height in spring (cm)	**3.83 ± 2.90**	**5.93 ± 4.23**
Inter-row vegetation height in summer (cm)	10.88 ± 7.43	10.13 ± 8.55
Row vegetation height in summer (cm)	8.90 ± 12.30	6.33 ± 4.84
Vegetation cover in spring (%) in inter-rows	82.38 ± 19.44	80.84 ± 20.03
Vegetation cover in summer (%) in inter-rows	**61.70 ± 22.85**	**40.36 ± 25.43**
Bare ground cover in spring (%) in inter-rows	12.59 ± 19.59	11.09 ± 14.74
Bare ground cover in summer (%) in inter-rows	**11.75 ± 10.31**	**20.23 ± 11.14**

**Table 2:** Treatment variables of vineyards with different cover-crop management (mean ± standard deviation). Bold numbers display variables that are significantly different among species-poor, species-rich, and spontaneous vegetation cover. Tillage frequency differs in neighboring inter-rows; therefore, management measures were displayed for the inter-row in which vegetation data was collected in spring and summer.

	Species-Poor	Species-Rich	Spontaneous
Tillage frequency in inter-rows	1.00 ± 1.21	1.28 ± 1.38	0.83 ± 0.98
Tillage frequency in rows	**2.33 ± 0.89**	**3.29 ± 1.2**	**1.83 ± 1.17**
Years without tillage in inter-rows	**1.42 ± 1.38**	**2.29 ± 1.14**	**1.33 ± 1.37**
Mulching frequency in inter-rows	2.58 ± 1.24	1.71 ± 1.07	1.83 ± 0.75
Rolling frequency in inter-rows	**0.42 ± 0.79**	**1.07 ± 1.38**	**–**
Inter-row vegetation height in spring (cm)	**16.17 ± 13.62**	**7.89 ± 3.09**	**6.63 ± 2.05**
Row vegetation height in spring (cm)	4.45 ± 2.98	3.58 ± 3.07	4.72 ± 2.32
Inter-row vegetation height in summer (cm)	10.14 ± 8.54	11.18 ± 8.27	8.98 ± 4.33
Row vegetation height in summer (cm)	5.31 ± 3.04	9.38 ± 14.19	9.19 ± 5.99
Vegetation cover in spring in inter-rows (%)	75.79 ± 25.19	86.56 ± 8.76	75.87 ± 28.24
Vegetation cover in summer in inter-rows (%)	44.48 ± 24.88	51.98 ± 29.07	49.14 ± 22.15
Bare ground cover in spring in inter-rows (%)	13.75 ± 21.40	8.31 ± 7.64	13.06 ± 17.74
Bare ground cover in summer in inter-rows (%)	14.44 ± 9.79	16.23 ± 12.83	18.80 ± 10.13
Herbicide applications in rows	**0.42 ± 0.67**	**–**	**–**

**Table 3:** Model outputs for predation rates of *L. botrana* eggs and pupae. Significant *p*-values are displayed in bold (degrees of freedom (df) = 26).

	Management (Integrated/Organic)		Implementation of Cover-Crops (Species-Poor/Species-Rich/Spontaneous)		Proportion of Woody Seminatural Habitats	
	χ²	*p*	χ²	*p*	χ²	*p*
*L. botrana* eggs	1.69	0.193	1.28	0.528	0.01	0.918
*L. botrana* pupae	11.76	**<0.001**	6.46	**0.040**	0.52	0.469

**Table 4:** Number of predator occurrences and pest control potential of camera-surveilled *L. botrana* sentinel cards in one selected vineyard pair. Nocturnal events are in parentheses.

	Individuals Captured on Photos		Individuals Involved in Predation or Parasitism		Predated *L. botrana* Pupae		Predation or Parasitism Incidents on *L. botrana* Eggs	
	Organic	Integrated	Organic	Integrated	Organic	Integrated	Organic	Integrated
**Acari**								
Anystidae	–	2 (0)	–	2 (0)	–	–	–	2 (0)
Trombidiidae	–	2 (1)	–	2 (1)	–	–	–	2 (1)
**Araneae**								
cf. *Cheiracanthium* sp.	7 (7)	9 (9)	2 (2)	3 (3)	–	–	2 (2)	3 (3)
cf. *Drassodes* sp.	–	1 (1)	–	–	–	–	–	–
cf. *Ebrechtella tricuspidata*	2 (0)	–	1 (0)	–	–	–	1 (0)	–
cf. *Marpissa muscosa*	–	3 (0)	–	–	–	–	–	–
cf. *Philodromus* sp.	1 (1)	–	1 (1)	–	–	–	1 (1)	–
cf. *Pseudicius encarpatus*	–	2 (0)	–	–	–	–	–	–
cf. Salticidae	1	6 (0)	–	–	–	–	–	–
*Cheiracanthium* sp.	6 (6)	–	1 (1)	–	1 (1)	–	–	–
Salticidae	1 (0)	–	1 (0)	–	–	–	1 (0)	–
*Salticus* sp.	1 (0)	–	–	–	–	–	–	–
**Coleoptera**								
Carabidae	6 (6)	2 (2)	2 (2)	2 (2)	7 (7)	4 (4)	–	–
Coccinellidae	–	1 (0)	–	–	–	–	–	–
Curculionidae	1 (0)	–	–	–	–	–	–	–
sp.	1 (0)	1 (0)	–	–	–	–	–	–
**Dermaptera**								
*Forficula auricularia*	38 (36)	57 (51)	16 (15)	34 (30)	16 (12)	52 (46)	9 (7)	8 (6)
**Diptera**								
sp.	13 (1)	6 (1)	–	–	–	–	–	–
**Hymenoptera**								
Formicidae	95 (24)	118 (22)	46 (20)	13 (2)	6 (1)	–	27 (9)	13 (2)
parasitic wasp	17 (16)	21 (15)	16 (16)	15 (11)	–	–	16 (16)	15 (11)
**Lepidoptera**								
sp.	–	1 (0)	–	–	–	–	–	–
**Neuroptera**								
Chrysopidae (adult)	–	1 (1)	–	1 (1)	–	–	–	1 (1)
Chrysopidae (larvae)	2 (2)	–	2 (2)	–	–	–	2 (2)	–
**Opiliones**								
sp.	1 (1)	–	–	–	–	–	–	–
**Orthoptera**								
*Leptophytes albovittata*	–	1 (1)	–	–	–	–	–	–
*Meconema meridionale*	–	2 (2)	–	2 (2)	–	9 (9)	–	–
*Meconema thalassinum*	–	2 (2)	–	2 (2)	–	3 (3)	–	–
*Oecanthus pellucens*	1 (1)	–	–	–	–	–	–	–
*Phaneroptera falcata*	7 (6)	–	5 (4)	–	15 (13)	–	–	–
*Phaneroptera nana*	4 (4)	–	3 (3)	–	13 (13)	–	–	–
*Tettigonia viridissima*	1 (1)	4 (4)	1 (1)	3 (3)	5 (5)	10 (10)	–	–

## Data Availability

All data are provided in the article and the Supplementary Materials.

## Supplementary Materials

The following are available online at https://www.mdpi.com/2075-4450/12/3/220/s1: Table S1. Detailed vineyard management and predation data for 32 studied Austrian vineyards.
