# Supplementary material for: Organic Farming and Cover-Crop Management Reduce Pest Predation in Austrian Vineyards
Source: Insects. 2021 Mar 4;12(3):220. doi: 10.3390/insects12030220 (PMC7999927; doi:10.3390/insects12030220)
Supplement: Supplementary file 1 [file insects-12-00220-s001.pdf]

Supplementary Table S1: Detailed vineyard management and predation data for 32 studied Austrian vineyards.

vineyard pair	farming type	inter-row management	aAPTLc	total number of applications	copper applications	sulphur applications	applications with synthetic fungicides	insecticide applications	acaricide applications	compost fertiliser (m³ / ha)	herbicide applications in rows	artificial fertiliser (kg / ha)	years without tillage	tillage frequency in inter-rows	mulching frequency in inter-rows	rolling frequency in inter-rows
1	conventional	species-rich cover-crop	47.68	8	2	4	8	1	0	0	0	0	3	0	2	3
1	organic	species-rich cover-crop	20.53	12	12	12	0	0	0	12.5	0	0	4	0	3	0
2	conventional	species-rich cover-crop	47.68	8	2	4	8	1	0	0	0	0	3	0	2	3
2	organic	species-rich cover-crop	13.98	9	7	9	0	0	0	0	0	0	3	0	0	1
3	conventional	species-rich cover-crop	8.07	11	2	8	11	0	0	0	2	0	2	3	1	3
3	organic	spontaneous vegetation	20.27	7	5	7	0	0	0	0	1	0	0	2	2	0
4	conventional	species-rich cover-crop	8.07	11	2	8	11	0	0	0	1	0	2	3	1	3
4	organic	species-rich cover-crop	5.25	10	7	8	0	0	1	10	0	0	2	0	1	0
5	conventional	species-poor cover-crop	14.46	6	1	2	3	0	0	0	1	0	3	0	3	0
5	organic	species-poor cover-crop	16.84	9	9	8	0	0	0	10	0	0	4	0	3	0
6	conventional	species-poor cover-crop	1.71	5	1	3	4	0	0	0	0	0	0	0	2	0
6	organic	species-rich cover-crop	19.68	8	8	8	0	0	0	0	0	0	3	2	3	0
7	conventional	species-poor cover-crop	1.71	5	1	3	4	0	0	0	0	0	0	0	2	0
7	organic	species-rich cover-crop	19.68	8	8	8	0	0	0	0	0	0	3	2	3	0
8	conventional	spontaneous vegetation	0.37	2	0	2	2	0	0	0	0	0	3	0	2	0
8	organic	species-rich cover-crop	19.68	8	8	8	0	0	0	0	0	0	3	0	3	0
9	conventional	species-poor cover-crop	1.71	5	1	3	4	0	0	0	0	0	0	0	2	0
9	organic	species-poor cover-crop	32.56	12	11	12	0	0	0	0	0	0	2	1	1	0
10	conventional	spontaneous vegetation	0.79	6	0	4	6	0	0	10	0	0	3	0	1	0
10	organic	spontaneous vegetation	7.10	7	7	7	0	0	0	10	0	0	0	2	2	0
11	conventional	spontaneous vegetation	7.96	6	2	2	6	0	0	0	0	0	1	1	1	0
11	organic	species-poor cover-crop	26.26	10	9	9	0	0	0	0	0	0	2	1	4	0
12	conventional	species-poor cover-crop	6.66	6	1	3	6	0	0	0	0	0	1	3	4	1
12	organic	species-poor cover-crop	26.26	10	9	9	0	0	0	0	0	0	0	0	4	0
13	conventional	species-rich cover-crop	8.09	6	1	5	6	0	0	0	0	150	0	3	2	0
13	organic	species-poor cover-crop	21.23	11	10	10	0	0	0	0	0	0	1	2	1	2
14	conventional	species-rich cover-crop	8.09	6	1	5	6	0	0	0	0	150	0	3	2	0
14	organic	species-poor cover-crop	21.23	11	10	10	0	0	0	0	0	0	1	2	1	2
15	conventional	spontaneous vegetation	1.07	4	0	4	4	0	0	0	0	0	1	0	3	0
15	organic	species-rich cover-crop	22.86	11	11	11	0	0	0	0	0	0	2	2	0	2
16	conventional	species-poor cover-crop	5.94	6	3	6	3	0	1	0	0	200	3	3	4	0
16	organic	species-rich cover-crop	25.70	12	11	11	0	0	0	10	0	0	2	0	1	0

vineyard pair	farming type	inter-row management	tillage frequency in rows	tillage frequency in rows	vegetation cover in spring (%) in inter-rows	bare ground cover in spring (%) in inter-rows	vegetation cover in summer (%) in inter-rows	bare ground cover in summer (%) in inter-rows	inter-row vegetation height in spring (cm)	row vegetation height in spring (cm)	inter-row vegetation height in summer (cm)	row vegetation height in summer (cm)	initial number of pupae	predated number of pupae	initial number of eggs	predated number of eggs
1	conventional	species-rich cover-crop	4	0	90	7.5	94.75	3.5	10.4	18	26.1	4.4	100	79	1029	967
1	organic	species-rich cover-crop	3	0	95.75	2.75	62.5	6.5	10.4	0	9.4	7	100	69	1053	957
2	conventional	species-rich cover-crop	4	0	98.25	0.75	50	20	11.6	4.8	11.5	16.5	100	84	896	793
2	organic	species-rich cover-crop	1	0	93.5	2	75	0	10.3	10.8	33.7	51.5	100	76	948	838
3	conventional	species-rich cover-crop	5	0	88.75	7.75	15.25	33.75	7.3	6.1	3.9	2.3	100	66	1002	744
3	organic	spontaneous vegetation	3	0	96	3.5	52.5	16.25	6.1	6.2	4.5	1.1	100	73	924	734
4	conventional	species-rich cover-crop	5	0	96.5	3	36.25	35	8.3	5.6	8.7	1.7	100	90	1189	1064
4	organic	species-rich cover-crop	5	0	67.5	27.75	88.75	8.75	4.1	1.1	14.2	6	100	83	1016	821
5	conventional	species-poor cover-crop	0	2	59	5	8.75	15	9.8	3.9	3.2	8.6	100	86	913	799
5	organic	species-poor cover-crop	3	0	96.5	2	85	2.75	12	2.3	17.4	4	100	91	944	816
6	conventional	species-poor cover-crop	2	1	36.5	16.75	55	20	11.3	11	6.9	8.7	100	81	922	837
6	organic	species-rich cover-crop	3	0	73.75	7.25	77.5	5.5	12.7	5.6	6.9	6.1	100	84	796	688
7	conventional	species-poor cover-crop	2	1	95.25	2.25	32.5	10	10.3	3.1	12.2	5.9	100	83	1162	1108
7	organic	species-rich cover-crop	3	0	90.5	0.75	91	1.5	7.1	3	10.2	2.3	100	82	1065	1002
8	conventional	spontaneous vegetation	1	0	90	1.75	87	4	9.8	4.3	13.1	8.1	100	89	958	830
8	organic	species-rich cover-crop	3	0	91	2	61.25	16.25	5.1	1.9	17.8	9.5	100	60	929	815
9	conventional	species-poor cover-crop	2	1	89.75	10.25	40	16.25	43.1	7.8	34.3	5.2	100	91	871	734
9	organic	species-poor cover-crop	3	0	79.5	1	85	5.5	43.1	5.8	8.8	9.5	100	70	1014	922
10	conventional	spontaneous vegetation	2	0	93.75	3.75	72.5	10	6.4	9.7	5.8	11.4	100	90	993	883
10	organic	spontaneous vegetation	2	0	74	22.5	57.5	26.25	8.8	6.4	8.8	8.9	100	80	1111	847
11	conventional	spontaneous vegetation	0	0	87.75	11	31.25	41.25	7.6	6.9	5.2	15.8	100	95	1021	771
11	organic	species-poor cover-crop	3	0	99.25	0.75	35	31.25	26.3	2	6.3	1.9	100	71	958	857
12	conventional	species-poor cover-crop	2	0	80	10	21.25	21.25	6.4	3.3	5.4	3.1	100	88	1112	1017
12	organic	species-poor cover-crop	3	0	98.75	1	71.25	0	9.3	7	6.4	9.4	100	72	1086	922
13	conventional	species-rich cover-crop	3	0	84.5	14.25	16.25	13.75	12.4	0.2	3.6	2.6	100	95	1063	914
13	organic	species-poor cover-crop	3	0	66.25	33.75	32.5	5	8.4	0.4	3.9	1.5	100	72	1039	822
14	conventional	species-rich cover-crop	3	0	83.75	12.5	32.5	32.5	4	2.1	6.9	2.1	100	80	1040	799
14	organic	species-poor cover-crop	3	0	22.5	74.75	45	21.25	6.5	2.2	6.9	2.2	100	73	1087	832
15	conventional	spontaneous vegetation	3	0	33.5	63.5	30	22.5	5.1	3.4	5.2	1.2	100	98	1238	1149
15	organic	species-rich cover-crop	2	0	82.5	12	57.5	13.75	4.6	2.5	13.2	19.4	100	95	1353	1066
16	conventional	species-poor cover-crop	2	0	86.25	7.5	22.5	25	7.5	4.6	10	3.7	100	71	1219	1030
16	organic	species-rich cover-crop	2	0	90.75	7.75	10	27.5	8.4	4	5.7	2.1	100	67	1143	933
